# Associations of metabolic heterogeneity of obesity with the progression of cardiometabolic multimorbidity—a nationwide prospective cohort study

**DOI:** 10.3389/fnut.2025.1617929

**Published:** 2025-08-21

**Authors:** Nian Cai, Lin Zhang, Shuai Ding, Xiaofang Tian, Li Mo, Bohai Yu

**Affiliations:** Shenzhen Hospital (Futian) of Guangzhou University of Chinese Medicine, Shenzhen, Guangdong, China

**Keywords:** obesity, metabolic status, transitions, heterogeneity, cardiometabolic multimorbidity

## Abstract

**Background:**

Previous studies have demonstrated that both obesity and metabolic heterogeneity impact cardiovascular disease. However, the effect of different body mass index (BMI)-metabolic phenotypes on the progression of cardiometabolic multimorbidity (CMM) remains unclear.

**Methods:**

This study utilized baseline data from the China Health and Retirement Longitudinal Study (CHARLS) in 2011, enrolling 5,850 participants for a longitudinal cohort analysis. Laboratory data from 2015 were used to assess 4,471 participants and evaluate the association between BMI-metabolic phenotype transitions (2011–2015) and the incidence of CMM. Participants were categorized into four BMI-metabolic phenotype groups: metabolically healthy normal weight (MHNW), metabolically healthy overweight/obesity (MHOO), metabolically unhealthy normal weight (MUNW), and metabolically unhealthy overweight/obesity (MUOO). Logistic regression models adjusted for potential confounders were applied to analyze the relationship between BMI-metabolic phenotypes, their dynamic changes, and CMM incidence.

**Results:**

Among the 5,850 participants, 562 (11.15%) developed CMM during the follow-up period. Both overweight/obesity and metabolically unhealthy status significantly accelerated CMM progression. The MUOO group exhibited the highest risk (OR = 3.31, 95% CI: 2.60–4.24; *p* < 0.001), followed by the MUNW (OR = 1.91, 95% CI: 1.47–2.47; *p* < 0.001) and MHOO groups (OR = 1.89, 95% CI: 1.30–2.69; *p* = 0.001), compared to the MHNW group. Further analysis revealed that changes in metabolic status had a greater impact on CMM risk than changes in BMI alone, with metabolic transitions in individuals with obesity being particularly associated with the onset of CMM.

**Conclusion:**

Worsening metabolic health and obesity significantly increase the risk of CMM. Notably, metabolic health plays a more critical role than obesity in predicting CMM incidence. This study highlights the importance of maintaining and improving metabolic health and suggests personalized obesity management strategies based on metabolic status to reduce CMM risk.

## Introduction

Cardiometabolic multimorbidity (CMM) is defined as the coexistence of two or more cardiometabolic diseases (CMD), such as type 2 diabetes (T2DM), stroke, or heart disease (1). Compared to individuals with a single CMD, those with CMM face higher mortality risks, with an average reduction in life expectancy of 12–15 years at age 60 (2). Furthermore, individuals with a single CMD or CMM are 1.41 times and 1.89 times more likely, respectively, to experience heightened psychological stress compared to those without CMD (3). The prevalence of CMM has been steadily rising worldwide in recent years. According to the World Health Organization (WHO), approximately 17 million deaths annually are attributable to CMM, accounting for over 30% of global mortality. A study of 500,000 individuals aged 30–79 years in China reported a CMM prevalence of 6.0% (4). Given the substantial disease burden of CMM, early identification of individuals at high risk may help prevent progression of the disease.

Obesity has become a global epidemic, particularly prevalent among middle-aged and older adults. Clinically, obesity is recognized as a chronic systemic disease, characterized by the abnormal or excessive accumulation of adipose tissue, which leads to structural and functional alterations in specific organs, tissues, or the body as a whole (5). Globally, over 1.9 billion adults are estimated to be overweight, with 650 million categorized as obese. Over the past 40 years, the prevalence of obesity has nearly doubled (6). Obesity is frequently accompanied by metabolic abnormalities; the resulting dysregulation is considered a key driver in the onset and progression of cardiovascular disease and diabetes, and is associated with a markedly increased risk of mortality (7).

In recent years, attention has turned to a specific subgroup of individuals with high fat mass but standard metabolic profiles, classified as metabolically healthy overweight/obesity (MHOO). Conversely, individuals with obesity and metabolic abnormalities are categorized as metabolically unhealthy overweight/obesity (MUOO) (8, 9). Similarly, heterogeneity in metabolic health is observed among individuals with normal body weight, leading to classifications as metabolically healthy normal weight (MHNW) and metabolically unhealthy normal weight (MUNW) (10). Individuals with different BMI-metabolic phenotypes may exhibit distinct disease outcomes, underscoring the importance of developing targeted prevention strategies tailored to these specific phenotypes. Emerging evidence suggests that transitions in BMI-metabolic phenotypes are dynamic and that shifts from metabolically healthy to unhealthy phenotypes are strongly associated with increased risks of cardiovascular diseases and diabetes (11–13).

The impact of different BMI-metabolic phenotypes on the progression of CMM remains poorly understood. Moreover, metabolic health is often unstable and may change over time, yet the specific effects of such transitions on the progression of CMM remain unclear. To address these gaps, this study utilized data from the China Health and Retirement Longitudinal Study (CHARLS) to investigate the influence of metabolic status on CMM risk in individuals with varying body weight. Additionally, we examined the relationship between transitions in BMI and metabolic phenotypes and the progression of CMM.

## Methods

### Study design and participants

The data for this study were obtained from the CHARLS, a nationwide survey targeting middle-aged and older adults in China. The survey participants were randomly selected individuals aged 45 years and above from sampled households. The CHARLS project conducted its baseline survey in 2011–2012, followed by subsequent waves in 2013 (Wave 2), 2015 (Wave 3), 2018 (Wave 4), and 2020 (Wave 5). During each follow-up, physical measurements were collected, and blood samples were obtained every two follow-up cycles. To ensure representativeness, the baseline survey covered 450 villages or neighborhoods from 150 counties or districts nationwide. In 2011, CHARLS successfully interviewed 17,708 individuals from 10,257 households, representing the middle-aged and older adult population in China. The CHARLS study protocol was approved by the Ethical Review Committee of Peking University, China, and written informed consent was obtained from all participants.

Samples used in this study were obtained from the China Center for Disease Control and Prevention and consisted of frozen plasma or whole blood stored at −80°C. Testing was conducted at the Center for Clinical Laboratory of Capital Medical University. During the analysis of the CHARLS study samples, quality control (QC) measures were implemented daily using QC samples. All QC results fell within the target range, remaining within two standard deviations of the mean QC control concentration, ensuring the reliability and consistency of the testing process.

**Figure 1:**
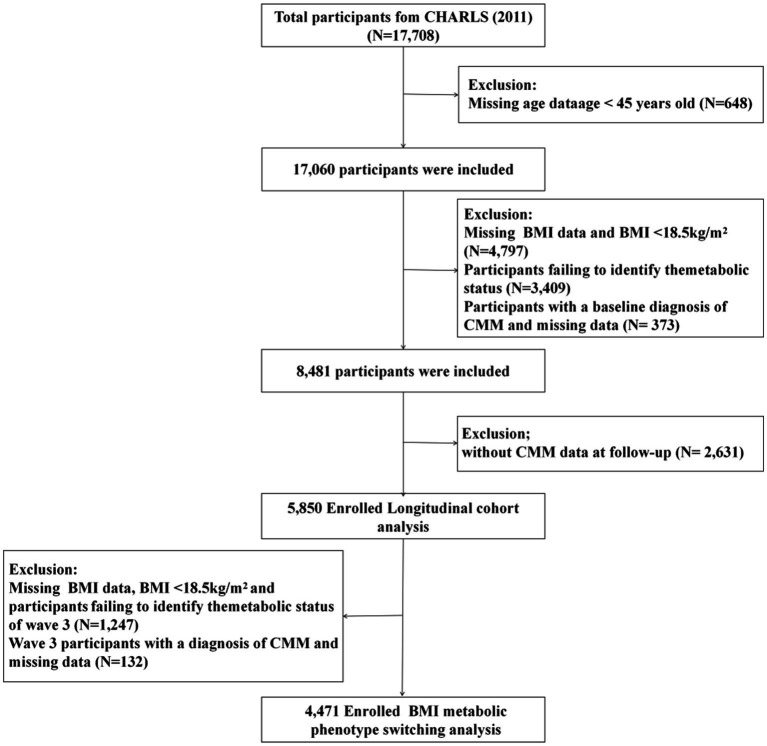
Flowchart of study participants.

This study utilized data from 2011 to 2020, excluding participants who were under 45 years old or had missing age data (*n* = 648), missing BMI data or BMI < 18.5 kg/m^2^ (*n* = 4,797), missing metabolic syndrome (MetS) data (*n* = 3,409), and those diagnosed with CMM or with missing CMM data at baseline (*n* = 373). Additionally, participants lost to follow-up (*n* = 2,631) were excluded, leaving a total of 5,850 individuals for longitudinal cohort analysis. To further evaluate the relationship between BMI-metabolic phenotype transitions and CMM incidence during 2011–2015, laboratory data from 2015 were used. Participants with missing BMI data, BMI < 18.5 kg/m^2^, or missing MetS data in Wave 3 (*n* = 1,247), as well as those diagnosed with CMM or with missing CMM data in Wave 3 (*n* = 132), were excluded. Ultimately, 4,471 participants were included in the final analysis (Figure 1).

To assess the potential for selection bias, we compared the distribution of sociodemographic characteristics, lifestyle factors, and metabolic indicators between included and excluded participants (Supplementary Table S1). The results showed relatively small differences between the two groups for most variables. However, statistically significant differences (*p* < 0.001) were observed in sex, marital status, educational level, and residence. Despite these differences, the overall distribution of key characteristics was broadly comparable, suggesting a limited risk of selection bias, although it cannot be entirely ruled out.

### Obesity and metabolic status definition

Obesity status was determined based on the Working Group on Obesity in China (WGOC) criteria (14), using BMI calculated from height and weight data. According to the BMI classification, individuals with a BMI of 18.5–25.0 kg/m^2^ are categorized as having a normal weight, while those with a BMI ≥ 25.0 kg/m^2^ are classified as being overweight or obese. Metabolic health was assessed based on the Adult Treatment Panel III (ATP III) criteria for Mets (15). Participants were considered metabolically unhealthy if they met any three of the following five components:

1. Abdominal obesity: Defined as a waist circumference (WC) ≥ 88 cm for women and ≥102 cm for men.
2. Elevated triglycerides (TG): TG levels ≥150 mg/dL or current treatment for elevated TG.
3. Low high-density lipoprotein cholesterol (HDL-C) levels: HDL-C levels <40 mg/dL for men and <50 mg/dL for women, or current treatment for low HDL-C.
4. Elevated blood pressure: Systolic blood pressure (SBP) ≥130 mm Hg and/or diastolic blood pressure (DBP) ≥85 mm Hg, or current antihypertensive treatment.
5. Elevated fasting plasma glucose (FPG): FPG levels ≥100 mg/dL, current treatment for diabetes, or a self-reported history of diabetes.

### Assessment of CMM events

Consistent with previous studies (2), we focused on three types of cardiometabolic diseases: diabetes, heart disease, and stroke. Participants were asked the following questions: “Have you ever been diagnosed with diabetes by a doctor?,” “Have you ever been diagnosed with heart disease (such as myocardial infarction, coronary heart disease, angina, congestive heart failure, or other heart conditions)?,” and “Have you ever been diagnosed with stroke (including cerebral infarction and cerebral hemorrhage)?” A response of “yes” to any of these questions was considered indicative of the corresponding condition. In addition to self-reported diabetes, participants were classified as having diabetes if they met any of the following criteria: (1) FPG ≥7.0 mmol/L; (2) random blood glucose ≥11.1 mmol/L; or (3) glycated hemoglobin (HbA1c) ≥6.5%, in accordance with the diagnostic criteria of the American Diabetes Association (16). CMM was defined as the coexistence of two or more of the following conditions: diabetes, heart disease, or stroke.

### Covariate assessments

Demographic and health-related data were collected through a structured questionnaire administered by trained professionals, including information on age, sex, residence (rural or urban), marital status (married or unmarried), and educational level (primary school, middle school, high school, or college and above). Health-related variables included self-reported smoking and drinking status (classified as never, former, or current), sleep duration, and self-reported physician-diagnosed medical conditions (diabetes, heart disease, stroke, and dyslipidemia). Laboratory tests included TG, HDL-C, FPG, HbA1c, and C-reactive protein (CRP).

### Statistical analysis

Chi-square tests (for categorical variables), Wilcoxon rank-sum tests (for continuous variables with non-normal distribution), and t-tests (for continuous variables with normal distribution) were used to assess differences between the normal and CMM groups. Continuous variables were described using means and standard errors, while categorical variables were described using frequencies and percentages. Three logistic regression models were used to assess the odds ratios (ORs) and 95% confidence intervals (CIs) of CMM in participants with different phenotypes. Three models were constructed: the crude model (unadjusted), Model 1 (adjusted for age, sex, education level, marital status, residence, smoking, and drinking), and the fully adjusted model (further adjusted for sleep duration, dyslipidemia, CRP, SBP, and DBP). A similar approach was used to analyze the relationship between obesity and metabolic status transitions and CMM incidence, using stable MHNW as the reference. Subgroup analysis was performed to explore whether the association between different BMI-metabolic phenotypes and CMM events varied across subgroups. To examine the dose–response relationship between blood pressure, blood glucose, WC, and CMM in metabolically healthy and unhealthy participants, restricted cubic spline (RCS) analysis was used. In the spline model, adjustments were made using Model 2. All statistical analyses were conducted using R software (version 4.2.1). A *p*-value < 0.05 was considered statistically significant.

## Results

### Baseline characteristics

After a 7-year follow-up, a total of 5,850 participants were included, among whom 652 (11.15%) were diagnosed with CMM. Generally, CMM patients were older, had a higher proportion of females, and had lower education levels. Additionally, CMM patients had lower smoking and drinking rates and relatively shorter sleep durations. Individuals with CMM were more likely to have hypertension and dyslipidemia (*p* < 0.001) (Supplementary Table S2). Among the 5,850 participants, 2,583 (44.15%) were in the MHNW group, 498 (8.51%) in the MHOO group, 1,291 (22.07%) in the MUNW group, and 1,478 (25.26%) in the MUOO group. Among the four BMI-metabolic phenotypes, the MUOO group had the highest average values for BMI, WC, SBP, DBP, HbA1c, FBG, TG, and CRP, the lowest average HDL-C, and the highest proportions of individuals with hypertension and dyslipidemia (all *p* < 0.001). Regarding CMM, the MUOO group had the highest incidence, followed by MUNW and MHOO, while the MHNW group had the lowest incidence of CMM (Table 1).

**Table 1:** Baseline characteristics and CMM events of the study population classified by BMI-metabolic phenotypes.

Variables	Total (*n* = 5,850)	MHNW (*n* = 2,583)	MHOO (*n* = 498)	MUNW (*n* = 1,291)	MUOO (*n* = 1,478)	*p*
Age (years)	57 (51, 63)	57 (51, 63)	54 (48, 60)	60 (54, 66)	57 (51, 63)	<0.001
Sex (*n*, %)						
Female	3,239 (55.37)	1,151 (44.56)	326 (65.46)	837 (64.83)	925 (62.58)	
Male	2,611 (44.63)	1,432 (55.44)	172 (34.54)	454 (35.17)	553 (37.42)	
Marital (*n*, %)						<0.001
Non-married	542 (9.26)	237 (9.18)	32 (6.43)	167 (12.94)	106 (7.17)	
Married	5,308 (90.74)	2,346 (90.82)	466 (93.57)	1,124 (87.06)	1,372 (92.83)	
Education (*n*, %)						<0.001
Below primary school	2,736 (46.78)	1,180 (45.68)	211 (42.37)	692 (53.64)	653 (44.18)	
Primary school	1,305 (22.31)	627 (24.27)	93 (18.67)	265 (20.54)	320 (21.65)	
Middle school	1,222 (20.89)	533 (20.63)	130 (26.10)	224 (17.36)	335 (22.67)	
High school and above	586 (10.02)	243 (9.41)	64 (12.85)	109 (8.45)	170 (11.50)	
Location (*n*, %)						<0.001
Village	1880 (32.14)	695 (26.91)	176 (35.34)	415 (32.15)	594 (40.19)	
City/Town	3,970 (67.86)	1888 (73.09)	322 (64.66)	876 (67.85)	884 (59.81)	
Smoking (*n*, %)						<0.001
Never smoker	3,682 (63.00)	1,397 (54.15)	365 (73.29)	884 (68.63)	1,036 (70.09)	
Former smoker	461 (7.89)	188 (7.29)	37 (7.43)	93 (7.22)	143 (9.68)	
Current smoker	1701 (29.11)	995 (38.57)	96 (19.28)	311 (24.15)	299 (20.23)	
Drinking (*n*, %)						<0.001
Never drinker	3,486 (59.61)	1,399 (54.18)	308 (61.85)	823 (63.75)	956 (64.73)	
Former drinker	459 (7.85)	194 (7.51)	32 (6.43)	105 (8.13)	128 (8.67)	
Current drinker	1903 (32.54)	989 (38.30)	158 (31.73)	363 (28.12)	393 (26.61)	
Sleep duration	7 (5, 8)	6 (5, 8)	7 (6, 8)	6 (5, 8)	7 (5, 8)	0.388
BMI (kg/m^2^)	23.4 (21.4, 26.0)	21.5 (20.2, 22.9)	26.5 (25.6, 28.2)	23.1 (21.8, 24.0)	27.4 (26.1, 29.2)	<0.001
WC (cm)	85.0 (78.8, 92.1)	79.0 (75.0, 83.6)	91.0 (86.0, 96.0)	85.8 (81.2, 89.4)	96 (91.0, 100.0)	<0.001
FPG (mg/dL)	102.06 (94.50, 112.32)	98.10 (91.80, 106.38)	96.30 (91.26, 103.09)	106.74 (100.22, 120.06)	107.64 (100.26, 119.61)	<0.001
HbAlc (%)	5.1 (4.9, 5.4)	5.1 (4.8, 5.3)	5.1 (4.9, 5.4)	5.2 (4.9, 5.5)	5.2 (5.0, 5.6)	<0.001
TG (mg/dL)	107.08 (75.22, 156.65)	83.19 (63.72, 110.62)	95.58 (71.68, 120.36)	150.89 (99.12, 207.09)	153.99 (107.08, 219.92)	<0.001
HDL (mg/dL)	49.10 (40.21, 59.54)	56.06 (47.94, 65.34)	53.35 (47.17, 60.70)	43.30 (36.34, 51.80)	40.98 (34.79, 48.33)	<0.001
CRP (mg/L)	0.99 (0.54, 2.01)	0.75 (0.45, 1.5)	0.96 (0.57, 1.82)	1.07 (0.57, 2.18)	1.44 (0.79, 2.78)	<0.001
Sbp (mm Hg)	126.33 (114.33, 141.00)	119.33 (110.00, 130.67)	119.67 (111.33, 129.00)	134.67 (120.67, 148.33)	136.33 (124.33, 149.33)	<0.001
Dbp (mm Hg)	75.00 (67.33, 83.33)	71.00 (64.33, 78.33)	73.33 (67.33, 79.67)	77.67 (70.33, 85.67)	81.00 (73.00, 89.00)	<0.001
Hypertension (*n*, %)						<0.001
No	4,372 (74.95)	2,307 (89.59)	452 (91.13)	846 (65.68)	767 (52.04)	
Yes	1,461 (25.05)	268 (10.41)	44 (8.87)	442 (34.32)	707 (47.96)	
Dyslipidemia (*n*, %)						<0.001
No	5,213 (90.28)	2,477 (96.91)	459 (93.48)	1,133 (88.93)	1,144 (78.73)	
Yes	561 (9.72)	79 (3.09)	32 (6.52)	141 (11.07)	309 (21.27)	
CMM (*n*, %)						<0.001
No	5,198 (88.85)	2,448 (94.77)	451 (90.56)	1,130 (87.53)	1,169 (79.09)	
Yes	652 (11.15)	135 (5.23)	47 (9.44)	161 (12.47)	309 (20.91)	

MHNW, metabolically healthy normal weight; MHOO, metabolically healthy overweight/obesity; MUNW, metabolically unhealthy normal weight; MUOO, metabolically unhealthy overweight/obesity; BMI, body mass index; WC, waist circumference; SBP, systolic blood pressure; DBP, diastolic blood pressure; HbA1c, glycated hemoglobin; FBG, fasting blood glucose; TG, triglyceride; HDL-C, high-density lipoprotein cholesterol; CRP, C-reactive protein; CMM, cardiometabolic multimorbidity.

### Associations of BMI-metabolic phenotypes with CMM incidence

**Figure 2:**
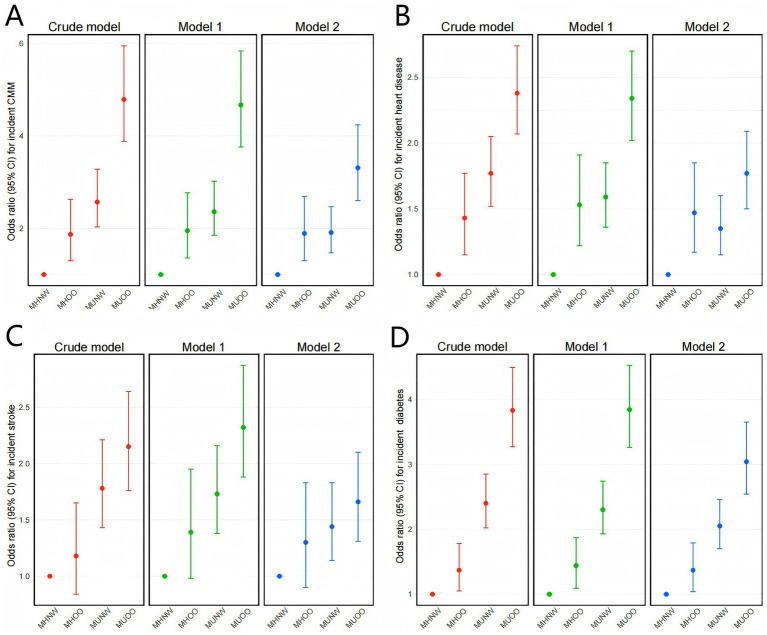
Associations of BMI-metabolic phenotypes with the CMM incidence. Crude model: Unadjusted. Model 1: Adjusted for age, sex, education, marital status, residence, smoking, and drinking. Model 2: Adjusted for the factors in model 1and sleep duration, dyslipidemia, CRP, SBP, and DBP.

The association between BMI-metabolic phenotypes and CMM was assessed in three models (crude model, model 1, and model 2), with the results as presented in Figure 2. Detailed information for all associations is provided in Supplementary Table S3. All models consistently indicated that both overweight/obesity and metabolically unhealthy status significantly increased the risk of CMM. After full adjustment, compared to MHNW, individuals with MHOO (OR = 1.89, 95% CI: 1.30–2.69; *p* = 0.001), MUNW (OR = 1.91, 95% CI: 1.47–2.47; *p* < 0.001), and MUOO (OR = 3.31, 95% CI: 2.60–4.24; *p* < 0.001) phenotypes exhibited a higher risk of CMM, with the MUOO group showing the highest risk, followed by MUNW and MHOO. A similar trend was observed in the analysis of stroke and diabetes; however, in the case of heart disease, after full adjustment, the MHOO group had a higher risk than the MUNW group.

### Subgroup analysis

**Figure 3:**
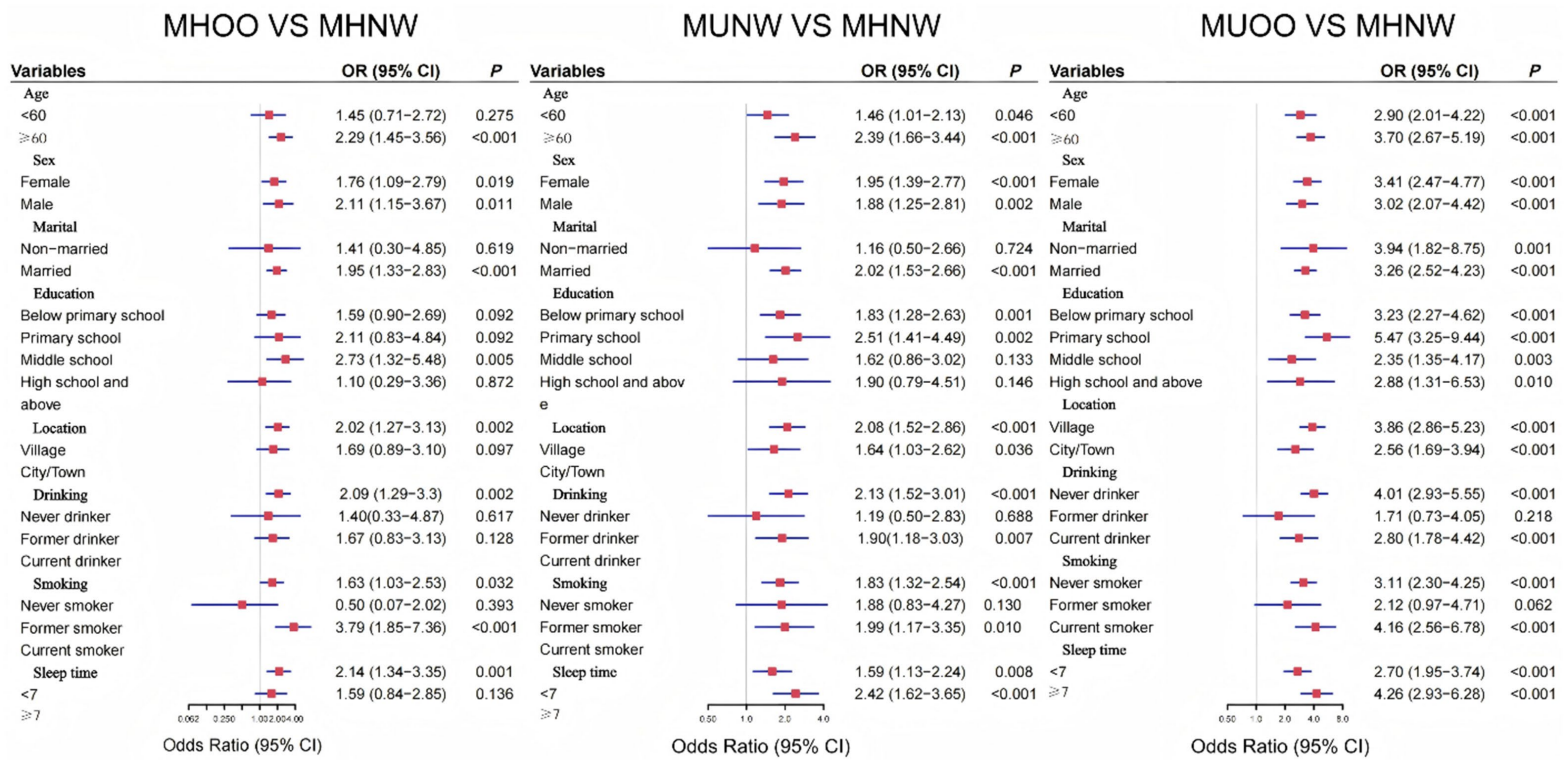
Associations of BMI-metabolic phenotypes with the CMM incidence among different subgroups. Adjusted for age, six, education, marital status, smoking, drinking, sleep duration, dyslipidemia, CRP, SBP, and DBP.

To investigate whether the association between different BMI-metabolic phenotypes and CMM events varies across subgroups, this study stratified participants based on their socioeconomic characteristics and performed subgroup analyses (Figure 3). The results revealed that, compared to MHNW, both overweight/obesity and metabolically unhealthy status increased the risk of CMM, independent of age, six, education level, smoking, drinking status, social interaction, and sleep duration (*p* > 0.05).

**Figure 4:**
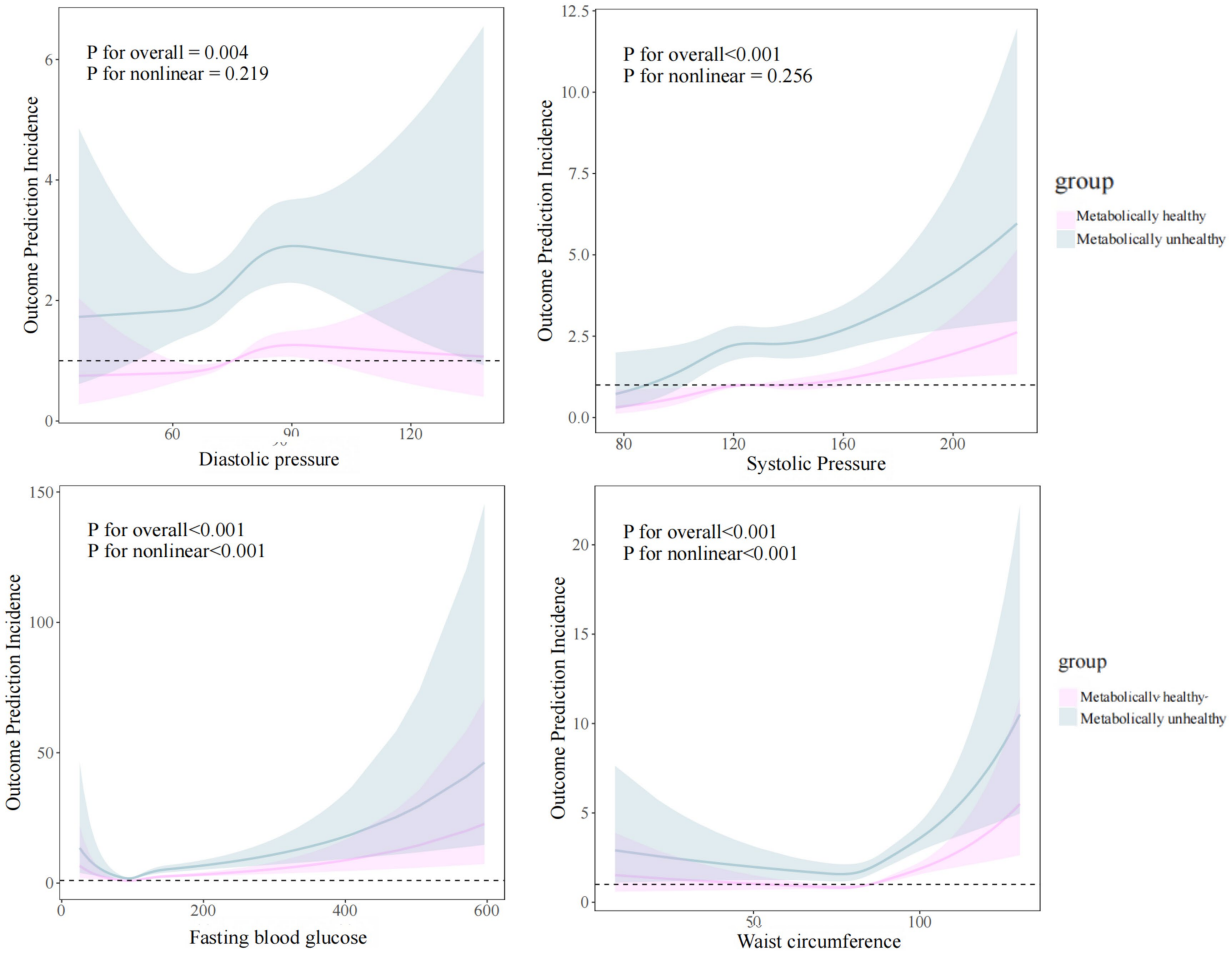
Restricted cubic spline model of the association of blood pressure, blood glucose, and waist circumference and CMM among the metabolically healthy and unhealthy groups. Adjusted for age, six, education, marital status, smoking, drinking, sleep duration, dyslipidemia, CRP, SBP, and DBP.

To further explore the nonlinear relationship, we fitted restricted cubic spline models. These models indicated that, in both metabolically healthy and metabolically unhealthy populations, the risk of CMM increased with blood pressure, blood glucose, and waist circumference. However, the risk was higher in the metabolically unhealthy group compared to the metabolically healthy group (Figure 4).

### Associations of BMI-metabolic phenotypes transitions with CMM incidence

**Figure 5:**
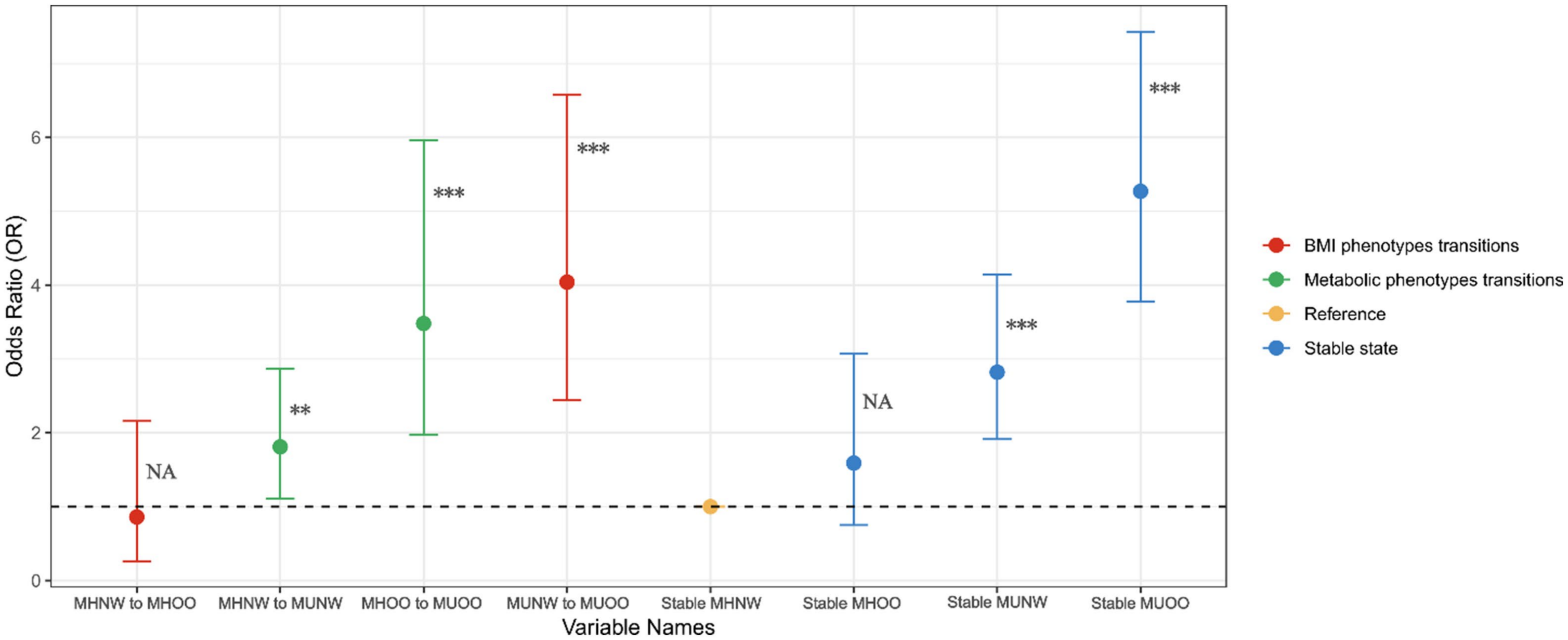
Associations of metabolic status transitions with the CMM incidence. Adjusted for age, sex, education, marital status, smoking, drinking, sleep duration, dyslipidemia, CRP, SBP, and DBP.

The changes in metabolic and obesity status over time are shown in Supplementary Table S4. Next, we investigated whether changes in metabolic health status affect the incidence of CMM. In the fully adjusted model, compared to the stable MHNW group (Figure 5), only the transition from MUNW to MUOO was significantly associated with an increased risk of CMM (OR: 4.40, 95% CI: 2.44–6.58, *p* < 0.001). When metabolic status changed, the transition from MHNW to MUNW (OR: 1.81, 95% CI: 1.11–2.87, *p* < 0.001) and from MHOO to MUOO (OR: 3.48, 95% CI: 1.97–5.96, *p* < 0.001) were both significantly associated with the occurrence of CMM. In stable states, the risk of CMM was significantly increased in the stable MUNW (OR: 2.82, 95% CI: 1.92–4.14, *p* < 0.001) and stable MUOO groups (OR: 5.27, 95% CI: 3.78–7.43, *p* < 0.001), whereas no significant differences were found in the stable MHOO group.

In the comparison between different metabolic states (Table 2), changes in BMI status did not show a significant association with the risk of CMM. However, when metabolically healthy individuals transitioned to an unhealthy metabolic state (MHNW to MUNW, MHOO to MUOO), the incidence of CMM significantly increased by 74% (OR: 1.74, 95% CI: 1.05–2.83, *p* = 0.028) and 176% (OR: 2.76, 95% CI: 1.18–6.85, *p* = 0.022), respectively. Conversely, when metabolically unhealthy individuals improved to a healthy metabolic state (MUNW to MHNW, MUOO to MHOO), the risk of CMM decreased by 36 and 71% (OR: 0.64, 95% CI: 0.38–1.05, *p* = 0.081; OR: 0.29, 95% CI: 0.14–0.55, *p* < 0.001). These results suggest that changes in metabolic status significantly impact the incidence of CMM. Further analysis explored whether obesity status plays a moderating role in the relationship between changes in metabolic health and changes in CMM risk. Compared to participants transitioning from MHNW to MUNW, those transitioning from MHOO to MUOO showed a 107% increase in CMM risk (OR: 2.07, 95% CI: 1.05–4.05, *p* = 0.033). In contrast, individuals transitioning from MUOO to MHOO had a similar risk of CMM as those transitioning from MUNW to MHNW (OR: 0.99, 95% CI: 0.42–2.23, *p* = 0.989).

**Table 2:** Intergroup comparisons for the CMM incidence among different BMI-metabolic phenotype transitions.

BMI-metabolic phenotype transitions	OR (95% CI)	*p*-value
BMI phenotypes transitions		
Stable MUOO	Reference	Reference
MUOO to MUNW	0.98 (0.61, 1.56)	0.948
Stable MUNW	Reference	Reference
MUNW to MUOO	1.38 (0.84, 2.25)	0.195
Stable MHOO	Reference	Reference
MHOO to MHNW	2.36 (0.58, 9.11)	0.211
Stable MHNW	Reference	Reference
MHNW to MHOO	0.94 (0.28, 2.41)	0.906
Metabolic phenotypes transitions		
Stable MHNW	Reference	Reference
MHNW to MUNW	1.74 (1.05, 2.83)	0.028
Stable MUNW	Reference	Reference
MUNW to MHNW	0.64 (0.38, 1.05)	0.081
Stable MHOO	Reference	Reference
MHOO to MUOO	2.76 (1.18, 6.85)	0.022
Stable MUOO	Reference	Reference
MUOO to MHOO	0.29 (0.14, 0.55)	<0.001
The impact of BMI on metabolic phenotype transitions		
MHNW to MUNW	Reference	Reference
MHOO to MUOO	2.07 (1.05, 4.05)	0.033
MUNW to MHNW	Reference	Reference
MUOO to MHOO	0.99 (0.42, 2.23)	0.989

Adjusted for age, sex, education, marital status, smoking, drinking, sleep duration, dyslipidemia, CRP, SBP, and DBP.

## Discussion

In this study, we explored the relationship between obesity-metabolic heterogeneity and the incidence of CMM. Compared to baseline MHNW, MHOO, MUNW, and MUOO all exhibited higher incidence rates of CMM during follow-up. Moreover, the dynamic changes in metabolic and obesity status significantly affected the risk of CMM. Specifically, the deterioration of obesity and metabolic health was associated with an increased incidence of CMM, while improvements in these states significantly reduced the risk of CMM. It is noteworthy that the dynamic changes in metabolic status were more effective in predicting CMM risk than changes in BMI alone. Additionally, obesity status played a moderating role in the relationship between metabolic health transitions and CMM risk, with the association between changes in metabolic health and CMM occurrence being more pronounced in individuals with obesity.

The prospective cohort results of this study are consistent with previous research. Several meta-analyses have shown that obesity combined with metabolic abnormalities significantly increases the risk of various age-related diseases, including T2DM, cardiovascular diseases (CVD), and cancers (17–19). In our study, we observed that metabolic abnormalities and obesity were both associated with the accelerated progression of CMM compared to MHNW. Notably, when metabolic health worsened, individuals with obesity had a significantly higher risk of developing CMM compared to those with normal weight.

A recent study based on a cohort of 9,393 Chinese adults in Beijing showed that individuals with MHOO had a higher risk of CVD (hazard ratio (HR): 1.91, 95% CI: 1.13–3.24) compared to those with MHNW (20). Similarly, another study based on the China Kadoorie Biobank (CKB), which included 458,246 participants, found that individuals with MUOO had a significantly increased risk of major vascular events (MVE), stroke, and ischemic heart disease (IHD) (21). In the field of diabetes, research has also revealed the significant impact of metabolic abnormalities and obesity on individual health. A prospective study of 432,763 Chinese adults found that after a median follow-up of 10.1 years, individuals with MHOO had an increased risk of developing diabetes compared to the MHNW group. However, their risk was significantly lower than that of the MUOO group (22). Additionally, individuals with MUNW had a higher risk of diabetes compared to those with MHNW, suggesting that normal weight does not necessarily mean metabolic health (23).

In summary, our findings further support the prevailing view that both obesity and changes in metabolic health jointly contribute to the development and progression of CMM. In particular, the deterioration of metabolic health is often accompanied by a cascade of metabolic disturbances—such as insulin resistance, ectopic fat accumulation, and dyslipidemia—that act synergistically to elevate the risk of cardiovascular and metabolic diseases substantially.

Furthermore, a single baseline assessment of metabolic health may not fully predict the long-term risk of CMM (21, 24). Some studies suggest that MHOO status may have a certain “protective effect” in the short term, with risks similar to those in the MHNW population (25). A cohort study demonstrated that under comparable high-fat dietary conditions, individuals with MHOO exhibited similar fat accumulation to those with MUOO. However, only the MUOO group experienced a decline in insulin sensitivity and worsening of several biochemical markers (26). This relative “protective effect” in MHOO individuals may be attributed to their preferential storage of excess energy in expandable subcutaneous adipose tissue, thereby limiting visceral and ectopic fat deposition, attenuating inflammatory responses, and preserving insulin sensitivity (27–29). Nonetheless, this metabolically healthy state is typically transient; longitudinal evidence indicates that most MHOO individuals eventually transition to the MUOO phenotype over time, substantially elevating their risk of developing cardiovascular disease, diabetes, and other chronic conditions (12, 30).

This study further supports this view, highlighting the importance of dynamic changes in both obesity and metabolic health status in the progression of CMM. Western studies consistently show that, over time, individuals with metabolic health tend to transition to a metabolically unhealthy state across all BMI categories, and this shift is closely associated with an increased risk of cardiovascular disease (30–32). Similarly, several large-scale studies in Asian populations have found that long-term exposure to a metabolically unhealthy state is associated with higher vascular risk (21, 33).

Our study further validates the significant impact of metabolic status changes on CMM, confirming that its importance exceeds that of obesity status, which is consistent with previous research (34). Specifically, compared to individuals who maintain their original metabolic health status, those whose metabolic health status changes significantly increase their risk of CMM; no such significant changes were observed in the BMI-change groups. This suggests that for metabolically unhealthy individuals, whether they gain or lose weight, it does not significantly affect the incidence of CMM. However, when metabolic health deteriorates, individuals with obesity experience a greater risk of CMM. The possible mechanism is that the deterioration of metabolic health may accelerate the progression of CMM by increasing pro-inflammatory responses and oxidative stress associated with visceral fat (35). Additionally, obesity may amplify these effects. Therefore, obesity plays a significant moderating role in the relationship between changes in metabolic health status and the risk of CMM. This effect is particularly pronounced in individuals with obesity, further emphasizing the critical role of metabolic health in the progression of chronic diseases.

The findings of our study have significant clinical and public health implications. By revealing the dynamic relationship between obesity metabolic phenotypes and the incidence of CMM, this study underscores the necessity of dynamically monitoring changes in metabolic health, beyond traditional BMI measures. In individuals with obesity, adipose tissue functions not merely as an energy reservoir but also as an active endocrine organ. If left uncontrolled, adipose tissue in obesity secretes a range of pro-inflammatory cytokines, which disseminate systemically via the circulation and induce a state of chronic low-grade inflammation (36). Particularly for those in the MHOO state, early identification and intervention of potential metabolic health issues may help prevent the transition to the MUOO state, which is crucial for CMM prevention.

In contrast, MUNW individuals, due to their normal body weight, are often overlooked in health management, which can potentially delay necessary interventions. One critical pathological mechanism of impaired metabolic health is the uneven distribution of fat. Studies show that the fat distribution characteristics of MUNW individuals differ from those of MUOO individuals, with a notable feature being lower lower-body fat in MUNW individuals (37). Previous research has demonstrated that lower-body fat, especially subcutaneous fat in the hips and thighs, not only stores excess energy but also improves insulin sensitivity and systemic lipid metabolism by reducing the release of free fatty acids and inflammatory factors (38, 39). Therefore, lower lower-body fat may lead to the redistribution of fat to visceral areas, exacerbating lipotoxicity and insulin resistance. Moreover, insufficient lower-body fat also impairs lipid metabolism regulation, potentially leading to dyslipidemia and chronic low-grade inflammation—important pathological mechanisms of cardiovascular disease (40). Recent genetic association studies have shown that genetically determined low gluteal-femoral fat and high abdominal fat are both linked to an increased risk of coronary heart disease and diabetes (41). Notably, MUNW individuals, due to their normal or low body weight, often mask their inherent risk of metabolic dysregulation, thereby increasing the challenges of health management. Therefore, traditional weight-centric screening strategies may be insufficient to uncover the underlying risks. A more comprehensive assessment incorporating metabolic indicators and measures of fat distribution is warranted to complement the limitations of BMI. It is crucial to clearly distinguish MUNW individuals from those with metabolically healthy normal weight to develop precise intervention measures and effectively prevent CMM.

For MUOO individuals, the Tübingen Lifestyle Intervention Program found that, although participants with MHOO or MUOO experienced a similar reduction in fat tissue mass, the improvement in insulin sensitivity among MUOO participants did not reach a level that would provide sufficient protection against type 2 diabetes and cardiovascular disease. In contrast, MHOO participants achieved protective levels of insulin sensitivity (42). Furthermore, baseline parameters revealed that increases in BMI and liver fat content were the strongest and most independent predictors of failure to transition from MUOO to metabolic health (43). This suggests that individuals with MUOO may require more substantial weight reduction to achieve metabolic health, particularly those with high adipose tissue dysfunction and hepatic fat accumulation. Given the greater severity of insulin resistance, chronic inflammation, and lipotoxicity typically observed in this group, interventions should extend beyond standard lifestyle modifications. More intensive and prolonged weight management strategies, potentially combined with pharmacotherapy, may be necessary to promote meaningful metabolic improvement.

Our study has the following limitations. First, there is currently no unified consensus on the definition of metabolic status, which may affect the comparability of the results. To minimize this limitation, we used widely accepted definitions of metabolic status. Second, this study primarily classified metabolic phenotypes based on BMI; however, the diversity of metabolic status and body measurement parameters suggests that relying solely on BMI may not accurately distinguish different metabolic phenotypes. Future research should consider incorporating additional indicators, such as fat distribution, waist-to-hip ratio, and muscle mass, to improve the accuracy of metabolic phenotype identification. Third, although we adjusted for a variety of covariates in the analysis, there may still be residual confounding or unmeasured potential influencing factors, such as dietary patterns, physical activity levels, and genetic susceptibility. These factors may somewhat impact the interpretability of the study’s conclusions. Finally, the sample in this study is derived from the middle-aged and elderly population in China, which may limit the generalizability of the results and their applicability to other populations.

## Conclusion

Our study found that, compared to metabolically healthy normal-weight individuals, dynamic changes in metabolic and obesity states significantly affect the risk of CMM. Regardless of obesity status, the transition from a metabolically healthy state to a metabolically unhealthy state significantly accelerates the progression of CMM, with this effect being particularly pronounced in individuals with obesity. Based on these findings, we emphasize the importance of regular monitoring and early intervention in metabolic health to reduce the risk of CMM and recommend a stratified management approach based on metabolic status in obesity.

## Data Availability

The original contributions presented in the study are included in the article/Supplementary material, further inquiries can be directed to the corresponding author.

## Glossary

CMM: Cardiometabolic multimorbidity
CHARLS: China Health and Retirement Longitudinal Study
MHNW: Metabolically healthy normal weight
MHOO: Metabolically healthy overweight/obesity
MUNW: Metabolically unhealthy normal weight
MUOO: Metabolically unhealthy overweight/obesity
WHO: World Health Organization
CMD: Mardiometabolic diseases
QC: Quality control
MetS: Metabolic syndrome
WGOC: Working Group on Obesity in China
ATP III: Adult Treatment Panel III
WC: Waist circumference
TG: Triglycerides
HDL-C: high-density lipoprotein cholesterol
SBP: systolic blood pressure
DBP: diastolic blood pressure
FPG: fasting plasma glucose
HbA1c: glycated hemoglobin
CRP: C-reactive protein
ORs: odds ratios
CIs: confidence intervals
RCS: restricted cubic spline
T2DM: Type 2 diabetes
CVD: cardiovascular diseases
CKB: China Kadoorie Biobank
MVE: major vascular events
IHD: ischemic heart disease
