# Supplementary material for: Associations of metabolic heterogeneity of obesity with the progression of cardiometabolic multimorbidity—a nationwide prospective cohort study
Source: Front Nutr. 2025 Aug 21;12:1617929. doi: 10.3389/fnut.2025.1617929 (PMC12408330; doi:10.3389/fnut.2025.1617929)
Supplement: Supplementary file 1 [file Table_1.docx]

Table S1 Baseline Characteristics of Included and Excluded Participants.

| Variables | Total (n = 17708) | Excluded (n = 11858) | Included (n = 5850) | *p* |
| --- | --- | --- | --- | --- |
| Age (years) | 58 (51, 65) | 58 (50, 66) | 58 (51, 63) | 0.051 |
| Sex (n, %) |  |  |  | < 0.001 |
| Female | 9227 (52.11) | 5988 (50.51) | 3239 (55.37) |  |
| Male | 8479 (47.89) | 5868 (49.49) | 2611 (44.63) |  |
| Marital (n, %) |  |  |  | < 0.001 |
| Non-married | 2258 (12.78) | 1716 (14.51) | 542 (9.26) |  |
| Married | 15417 (87.22) | 10109 (85.49) | 5308 (90.74) |  |
| Education (n, %) |  |  |  | < 0.001 |
| Below primary school | 8017 (45.43) | 5281 (44.77) | 2736 (46.78) |  |
| Primary school | 3727 (21.12) | 2422 (20.53) | 1305 (22.31) |  |
| Middle school | 3669 (20.79) | 2447 (20.74) | 1222 (20.89) |  |
| High school and above | 2233 (12.65) | 1647 (13.96) | 586 (10.02) |  |
| Location (n, %) |  |  |  | < 0.001 |
| Village | 7171 (40.50) | 5291 (44.62) | 1880 (32.14) |  |
| City/Town | 10537 (59.50) | 6567 (55.38) | 3970 (67.86) |  |
| Household income | 12000 (2400, 33600) | 12560 (2338, 34343) | 10600 (2500, 32100) | 0.211 |
| Smoking (n, %) |  |  |  | 0.233 |
| Never smoker | 10619 (62.81) | 6937 (62.7) | 3682 (63) |  |
| Former smoker | 1417 (8.38) | 956 (8.64) | 461 (7.89) |  |
| Current smoker | 4871 (28.81) | 3170 (28.65) | 1701 (29.11) |  |
| Drinking (n, %) |  |  |  | 0.278 |
| Never drinker | 10333 (58.9) | 6847 (58.55) | 3486 (59.61) |  |
| Former drinker | 1443 (8.23) | 984 (8.41) | 459 (7.85) |  |
| Current drinker | 5767 (32.87) | 3864 (33.04) | 1903 (32.54) |  |
| Sleep duration | 6 (5, 8) | 6 (5, 8) | 7 (5, 8) | 0.181 |
| HbAlc (%) | 5.1 (4.9, 5.4) | 5.1 (4.9, 5.4) | 5.1 (4.9, 5.4) | 0.447 |
| TG (mg/dL) | 106.2 (75.22, 156.65) | 106.2 (75.89, 155.76) | 107.08 (75.22, 156.65) | 0.719 |
| HDL (mg/dL) | 49.1 (39.82, 59.54) | 48.71 (39.82, 59.92) | 49.1 (40.21, 59.54) | 0.550 |
| Sbp (mmHg) | 127.33 (115, 142.67) | 128 (115.67, 144) | 126.33 (114.33, 141) | < 0.001 |
| Dbp (mmHg) | 75.33 (67.67, 84) | 75.67 (67.67, 84.33) | 75 (67.33, 83.33) | 0.056 |
| Hypertension (n, %) |  |  |  | 0.054 |
| No | 12935 (74.04) | 8563 (73.58) | 4372 (74.95) |  |
| Yes | 4535 (25.96) | 3074 (26.42) | 1461 (25.05) |  |
| Dyslipidaemia (n, %) |  |  |  | 0.688 |
| No | 15517 (90.15) | 10304 (90.08) | 5213 (90.28) |  |
| Yes | 1696 (9.85) | 1135 (9.92) | 561 (9.72) |  |

SBP, systolic blood pressure; DBP, diastolic blood pressure; HbA1c, glycated haemoglobin; FBG, fasting blood glucose; TG, triglyceride; HDL‐C, high‐density lipoprotein cholesterol
