# Supplementary material for: Associations of metabolic heterogeneity of obesity with the progression of cardiometabolic multimorbidity—a nationwide prospective cohort study
Source: Front Nutr. 2025 Aug 21;12:1617929. doi: 10.3389/fnut.2025.1617929 (PMC12408330; doi:10.3389/fnut.2025.1617929)
Supplement: Supplementary file 2 [file Table_2.docx]

Table S2 Baseline characteristics of the study population classified according to CMM events.

| Variables | Total (n = 5850) | No CMM (n = 5198) | CMM (n = 652) | *p* |
| --- | --- | --- | --- | --- |
| Age (year) | 57 (51, 63) | 57 (51, 63) | 59.5 (54, 64.25) | < 0.001 |
| Sex (n, %) |  |  |  | < 0.001 |
| Female | 3239 (55.37) | 2836 (54.56) | 403 (61.81) |  |
| Male | 2611 (44.63) | 2362 (45.44) | 249 (38.19) |  |
| Marital (n, %) |  |  |  | 0.989 |
| Non-married | 542 (9.26) | 481 (9.25) | 61 (9.36) |  |
| Married | 5308 (90.74) | 4717 (90.75) | 591 (90.64) |  |
| Education (n, %) |  |  |  | 0.797 |
| Below primary school | 2736 (46.78) | 2423 (46.62) | 313 (48.01) |  |
| Primary school | 1305 (22.31) | 1157 (22.26) | 148 (22.7) |  |
| Middle school | 1222 (20.89) | 1095 (21.07) | 127 (19.48) |  |
| High school and above | 586 (10.02) | 522 (10.04) | 64 (9.82) |  |
| Location (n, %) |  |  |  | 0.183 |
| Village | 1880 (32.14) | 1655 (31.84) | 225 (34.51) |  |
| City/Town | 3970 (67.86) | 3543 (68.16) | 427 (65.49) |  |
| Smoking (n, %) |  |  |  | < 0.001 |
| Never smoker | 3682 (63) | 3248 (62.56) | 434 (66.56) |  |
| Former smoker | 461 (7.89) | 386 (7.43) | 75 (11.5) |  |
| Current smoker | 1701 (29.11) | 1558 (30.01) | 143 (21.93) |  |
| Drinking (n, %) |  |  |  | 0.005 |
| Never drinker | 3486 (59.61) | 3070 (59.07) | 416 (63.9) |  |
| Former drinker | 459 (7.85) | 400 (7.7) | 59 (9.06) |  |
| Current drinker | 1903 (32.54) | 1727 (33.23) | 176 (27.04) |  |
| Sleep duration | 7 (5, 8) | 7 (5, 8) | 6 (5, 8) | 0.001 |
| BMI | 23.4 (21.4, 26) | 23.3 (21.2, 25.7) | 25.5 (22.7, 28) | < 0.001 |
| WC (cm) | 58.6 (52.23, 66.3) | 58.2 (52, 65.6) | 62.85 (55.07, 71.8) | < 0.001 |
| FPG (mg/dL) | 102.06 (94.5, 112.32) | 101.52 (94.14, 110.7) | 110.43 (99.18, 127.62) | < 0.001 |
| HbAlc (%) | 5.1 (4.9, 5.4) | 5.1 (4.9, 5.4) | 5.3 (5, 5.8) | < 0.001 |
| TG (mg/dL) | 107.08 (75.22, 156.65) | 103.54 (74.34, 152.22) | 130.1 (92.7, 197.58) | < 0.001 |
| HDL (mg/dL) | 49.1 (40.21, 59.54) | 49.87 (40.98, 59.92) | 45.04 (36.63, 53.06) | < 0.001 |
| CRP (mg/L) | 5.1 (4.9, 5.4) | 5.1 (4.9, 5.4) | 5.3 (5, 5.8) | < 0.001 |
| Sbp (mmHg) | 126.33 (114.33, 141) | 125.67 (113.67, 139.67) | 132.67 (120.5, 148.67) | < 0.001 |
| Dbp (mmHg) | 75 (67.33, 83.33) | 74.33 (67.33, 82.67) | 78.17 (70.33, 86.42) | < 0.001 |
| Hypertension (n, %) |  |  |  | < 0.001 |
| No | 4372 (74.95) | 4061 (78.38) | 311 (47.7) |  |
| Yes | 1461 (25.05) | 1120 (21.62) | 341 (52.3) |  |
| Dyslipidaemia (n, %) |  |  |  | < 0.001 |
| No | 5213 (90.28) | 4731 (92.28) | 482 (74.5) |  |
| Yes | 561 (9.72) | 396 (7.72) | 165 (25.5) |  |
| BMI-metabolic phenotypes (n, %) |  |  |  | < 0.001 |
| MHNW | 2583 (44.15) | 2448 (47.1) | 135 (20.71) |  |
| MHOO | 498 (8.51) | 451 (8.68) | 47 (7.21) |  |
| MUNW | 1291 (22.07) | 1130 (21.74) | 161 (24.69) |  |
| MUOO | 1478 (25.26) | 1169 (22.49) | 309 (47.39) |  |

MHNW, metabolically healthy normal weight; MHOO, metabolically healthy overweight/obesity; MUNW, metabolically unhealthy normal weight; MUOO, metabolically unhealthy overweight/obesity; BMI, body mass index; WC, waist circumference; SBP, systolic blood pressure; DBP, diastolic blood pressure; HbA1c, glycated haemoglobin; FBG, fasting blood glucose; TG, triglyceride; HDL‐C, high‐density lipoprotein cholesterol; CRP, C-reactive protein; CMM, cardiometabolic multimorbidity
