# Supplementary material for: Associations of metabolic heterogeneity of obesity with the progression of cardiometabolic multimorbidity—a nationwide prospective cohort study
Source: Front Nutr. 2025 Aug 21;12:1617929. doi: 10.3389/fnut.2025.1617929 (PMC12408330; doi:10.3389/fnut.2025.1617929)
Supplement: Supplementary file 3 [file Table_3.docx]

|  | Crude model |  | Model 1 |  | Model 2 |  |
| --- | --- | --- | --- | --- | --- | --- |
| CMM |  |  |  |  |  |  |
| MHNW | ref | ref | ref | ref | ref | ref |
| MHOO | 1.87 [1.30, 2.63] | <0.001 | 1.95 [1.36, 2.77] | <0.001 | 1.89 [1.30, 2.69] | 0.001 |
| MUNW | 2.57 [2.03, 3.28] | <0.001 | 2.36 [1.85, 3.02] | <0.001 | 1.91 [1.47, 2.47] | <0.001 |
| MUOO | 4.79 [3.88, 5.95] | <0.001 | 4.67 [3.76, 5.84] | <0.001 | 3.31 [2.60, 4.24] | <0.001 |
| *P* for trend |  | <0.001 |  | <0.001 |  | <0.001 |
| Stroke |  |  |  |  |  |  |
| MHNW | ref | ref | ref | ref | ref | ref |
| MHOO | 1.18 [0.84, 1.65] | 0.326 | 1.39 [0.98, 1.95] | 0.061 | 1.30 [0.90, 1.83] | 0.15 |
| MUNW | 1.78 [1.43, 2.21] | <0.001 | 1.73 [1.38, 2.16] | <0.001 | 1.44 [1.14, 1.83] | 0.002 |
| MUOO | 2.15 [1.76, 2.64] | <0.001 | 2.32 [1.88, 2.87] | <0.001 | 1.66 [1.31, 2.10] | <0.001 |
| *P* for trend |  | <0.001 |  | <0.001 |  | <0.001 |
| Heart disease |  |  |  |  |  |  |
| MHNW | ref | ref | ref | ref | ref | ref |
| MHOO | 1.43 [1.15, 1.77] | 0.001 | 1.53 [1.22, 1.91] | <0.001 | 1.47 [1.17, 1.85] | 0.001 |
| MUNW | 1.77 [1.52, 2.05] | <0.001 | 1.59 [1.36, 1.85] | <0.001 | 1.35 [1.15, 1.60] | <0.001 |
| MUOO | 2.38 [2.07, 2.74] | <0.001 | 2.34 [2.02, 2.70] | <0.001 | 1.77 [1.50, 2.09] | <0.001 |
| *P* for trend |  | <0.001 |  | <0.001 |  | <0.001 |
| Diabetes |  |  |  |  |  |  |
| MHNW | ref | ref | ref | ref | ref | ref |
| MHOO | 1.37 [1.05, 1.78] | 0.019 | 1.44 [1.09, 1.87] | 0.008 | 1.37 [1.04, 1.79] | 0.025 |
| MUNW | 2.40 [2.02, 2.85] | <0.001 | 2.30 [1.93, 2.74] | <0.001 | 2.05 [1.70, 2.46] | <0.001 |
| MUOO | 3.83 [3.27, 4.49] | <0.001 | 3.84 [3.26, 4.52] | <0.001 | 3.04 [2.54, 3.65] | <0.001 |
| *P* for trend |  | <0.001 |  | <0.001 |  | <0.001 |

Table S3 Associations of BMI-metabolic phenotypes with the CMM incidence.

Crude model: Unadjusted.

Model 1: Adjusted for age, gender, education, marital status, residence, smoking, and drinking.

Model 2: Adjusted for the factors in Model 1and sleep duration, dyslipidemia, CRP, SBP, and DBP.
