# Supplementary material for: Associations of metabolic heterogeneity of obesity with the progression of cardiometabolic multimorbidity—a nationwide prospective cohort study
Source: Front Nutr. 2025 Aug 21;12:1617929. doi: 10.3389/fnut.2025.1617929 (PMC12408330; doi:10.3389/fnut.2025.1617929)
Supplement: Supplementary file 4 [file Table_4.docx]

**Table S4.** Number and percentage for the transitions of BMI-metabolic phenotypes

| BMI-metabolic phenotypes at baseline | BMI-metabolic phenotypes at the second resurvey, n (%) | | | |
| --- | --- | --- | --- | --- |
|  | MHNW | MUNW | MHOO | MUOO |
| MHNW | 1289(28.8) | 88(2.0) | 296(6.6) | 85(1.9) |
| MUNW | 110(2.4) | 162(3.6) | 93(2.1) | 145(3.2) |
| MHOO | 291(6.5) | 73(1.6) | 508(11.3) | 119(2.7) |
| MUOO | 77(1.7) | 145(3.2) | 162(3.6) | 828(18.5) |

MHNW, metabolically healthy normal weight; MHOO, metabolically healthy overweight/obesity; MUNW, metabolically unhealthy normal weight; MUOO, metabolically unhealthy obese; The second resurvey is at wave 3 (2015) for the CHARLS.
